# Protein Kinase C-θ (PKC-θ) in Natural Killer Cell Function and Anti-Tumor Immunity

**DOI:** 10.3389/fimmu.2012.00187

**Published:** 2012-07-05

**Authors:** Alberto Anel, Juan I. Aguiló, Elena Catalán, Johan Garaude, Moeez G. Rathore, Julián Pardo, Martín Villalba

**Affiliations:** ^1^Departamento de Bioquímica y Biología Molecular y Celular, Universidad de ZaragozaZaragoza, Spain; ^2^INSERM-U1040, Institut de Recherche en BiothérapieMontpellier, France; ^3^ARAID/Gobierno de AragónZaragoza, Spain

**Keywords:** PKC-θ, NK cells, anti-tumor immunity, CTL

## Abstract

The protein kinase C-θ (PKCθ), which is essential for T cell function and survival, is also required for efficient anti-tumor immune surveillance. Natural killer (NK) cells, which express PKCθ, play a prominent role in this process, mainly by elimination of tumor cells with reduced or absent major histocompatibility complex class-I (MHC-I) expression. This justifies the increased interest of the use of activated NK cells in anti-tumor immunotherapy in the clinic. The *in vivo* development of MHC-I-deficient tumors is much favored in *PKC*θ^−/−^ mice compared with wild-type mice. Recent data offer some clues on the mechanism that could explain the important role of PKCθ in NK cell-mediated anti-tumor immune surveillance: some studies show that PKCθ is implicated in signal transduction and anti-tumoral activity of NK cells elicited by interleukin (IL)-12 or IL-15, while others show that it is implicated in NK cell functional activation mediated by certain killer-activating receptors. Alternatively, the possibility that PKCθ is involved in NK cell degranulation is discussed, since recent data indicate that it is implicated in microtubule-organizing center polarization to the immune synapse in CD4^+^ T cells. The implication of PKC isoforms in degranulation has been more extensively studied in cytotoxic T lymphocyte, and these studies will be also summarized.

## PKCθ in T Cells

The protein kinase C-θ (PKC-θ) was initially isolated as a PKC isoform expressed in T cells (Baier et al., 1993), although it was demonstrated afterward that its expression was not restricted to them (Isakov and Altman, 2002). Structurally, PKCθ is a member of the novel, Ca^2+^-independent, PKC subfamily (which also includes PKCδ, ε, and η). PKCθ rapidly translocated to the immunological synapse (IS), suggesting a central role in T lymphocyte signal transduction (Monks et al., 1997). Seminal discoveries by the group of Amnon Altman demonstrated that PKCθ was indeed essential in T cell activation through the triggering of key transcription factors such as AP-1 (activating protein-1; Baier-Bitterlich et al., 1996), nuclear factor-κB (NF-κB; Coudronniere et al., 2000), and nuclear factor of activated T cell (NF-AT; Altman et al., 2004). This was confirmed by two independent groups who developed two different strategies to knockout PKCθ mice (Sun et al., 2000; Pfeifhofer et al., 2003). These transcription factors control the expression of target genes implicated in proliferation, survival, and/or cytotoxicity. One of them, Fas Ligand (FasL), is also implicated in immune homeostasis maintenance through activation-induced cell death (AICD). PKCθ deficient mice show normal lymphocyte development but display a selective phenotype in their mature T cell compartment, characterized by impaired proliferation and interleukin (IL)-2 production in response to T cell receptor (TCR)/CD28 co-stimulation (Sun et al., 2000; Pfeifhofer et al., 2003). This phenotype is also linked to the regulation of genes essential for survival, such as B-cell lymphoma (Bcl)-2 family members (Bertolotto et al., 2000; Villalba et al., 2001b; Barouch-Bentov et al., 2005). The mechanism by which PKCθ contributes to the first steps of T cell activation has recently been uncovered. A motif in the V3 domain determines its localization to the IS through the direct interaction with the co-stimulation molecule CD28 (Kong et al., 2011). Other experts in this issue extensively describe the biochemical and functional aspects of PKCθ regulation in T cells.

## Natural Killer Cells

The appearance of clinically detectable tumors may be the result of the proliferation of cells that have developed sophisticated strategies to escape the immune response. Arguably, the most relevant is the total or selective loss of expression of the major histocompatibility complex class-I (MHC-I). MHC-I, known as human leukocyte antigen (HLA) in humans, mediates self-recognition and thus present endogenously synthesized antigens to CD8^+^ cytotoxic T lymphocytes (CTLs). Changes in MHC-I allow tumor cells to avoid CTLs and thereby the adaptive immune response (Aptsiauri et al., 2007). However, a small amount of surface MHC-I must be maintained, because its absence would make tumor cells targets of natural killer (NK) cells.

**Figure 1:**
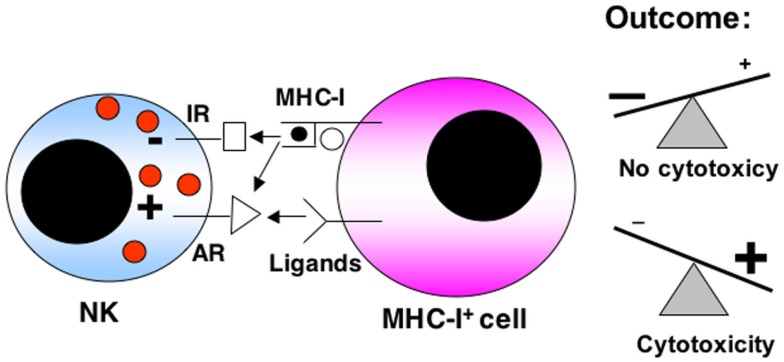
**Schematic representation of the balance between activating (AR) and inhibitory receptors (IR) in NK cell anti-tumoral function**. Receptors for MHC-I can be either inhibitory or activating. The expression levels of MHC-I or of the different ligands for the activating receptors on the target cell is of paramount importance in the final response. The final outcome between both types of signaling will determine if there is a cytotoxic response or not.

Natural killer cells are members of the innate immune system with natural cytotoxicity against tumor cells. This lymphocyte lineage produces cytokines and shows cytotoxicity and effector functions (Lanier, 2008; Velardi, 2008). NK cells predominantly target cells lacking MHC-I, which include transformed or virus-infected cells, which down-regulate MHC-I expression to avoid recognition by CTLs. Therefore the “missing self” hypothesis proposes that NK cells discriminate target cells from other healthy “self” cells based on MHC-I expression. However, it is now clear that NK cell activation depends on a complex signaling process mediated by activating and inhibitory receptors, being the functional outcome the final result of the different activating and inhibitory signals received (see the schematic representation shown in Figure 1).

One of the most potent activating NK cell receptor (killer-activating receptor, KAR) is CD16, a receptor for the Fc domain of IgG, also termed FcγRIII, which is responsible for antibody-dependent cell-mediated cytotoxicity (ADCC), one of the main physiological functions of NK cells. CD16 signals through its association with the immunoreceptor tyrosine-based activation motif (ITAM)-containing adaptors FcεR1γ and CD3ζ, which finally activate ZAP-70 and Syk protein tyrosine kinases (López-Botet et al., 2000; Lanier, 2008; KEGGpathway, 2011). Other human KARs that share a similar signal transduction machinery are NKp46 and NKp30. Influenza virus hemagglutinin (HA) and B7-H6 expressed on the surface of tumors, respectively, have been identified as ligands for these KAR, but it is also possible that other unidentified endogenous ligands exist.

NKp44, which ligand is also unknown, completes, together with NKp30 and NKp46, the so-called natural cytotoxicity receptors (NCR) family. However, NKp44 signals through a different ITAM-containing adaptor, DAP12, that also results in the activation of ZAP-70 and Syk. This signaling adaptor is shared by several activating receptors belonging to the killer cell immunoglobulin-like receptors (KIR) family: KIR2DS1 and KIR2DS4, which ligands are specific haplotypes of HLA-C, and KIR2DS2 and KIR3DS1, which ligands are still unknown. Finally, the DAP12 adaptor is also used by NKG2C, a member of a different family of NK cell receptors, the C-lectin type family. This activating receptor is expressed as a heterodimer with CD94 and its ligand is HLA-E.

Another activating receptor of the C-lectin type is NKG2D. This protein is expressed as a homodimer, and uses a different adaptor for signaling, DAP10. This adaptor does not contain ITAM domains in its cytoplasmic tail, but it contains one YINM motif, that, upon tyrosine phosphorylation recruits both PI3K and the Grb2-Vav1-Sos1 complex. The downstream signaling of this receptor does not require ZAP-70 and/or Syk activation, but results also in ERK and PKC activation. NKG2D ligands in humans are members of the MIC and ULBP families of proteins, which are expressed by virus-infected or tumoral cells.

Another family of activating receptors is the SLAM family, which includes CD244 (also known as 2B4), CRACC (CD319), and NTB-A. CD244 ligand is CD48, while CRACC and NTB-A promote homophilic interactions. These receptors posses TIYXX motifs in their cytoplasmic tails (also called immunoreceptor tyrosine-based switch motif, ITSM), which, upon tyrosine phosphorylation recruit the adaptors SAP or EAT2. If SAP is recruited, then the signals generated, including the activation of the protein tyrosine kinase Fyn, result in activation. However, if EAT2 is recruited, then signals are rather inhibitory.

Finally, two other activating receptors are expressed in human NK cells, DNAM-1, also known as CD226, and NKp80, also known as CLEC5C. It seems that DNAM-1 signaling is dependent on specific domains present in its own cytoplasmic tail (Shibuya et al., 1998). On the other hand, it has been recently demonstrated that NKp80 transmits signals through a semi-ITAM domain present in its cytoplasmic tail, activating directly Syk (Dennehy et al., 2011). The ligands for DNAM-1 are CD112 and CD155, molecules implicated in leukocyte adhesion, and the ligand for NKp80 is AICL, a very close homolog to NKp80 expressed on the surface of solid tumors.

In mice, NK cell activating receptors belong rather to the C-lectin family, normally forming homodimers on the surface of NK cells, and transmitting signals through the DAP12 adaptor. These are Ly49D, Ly49H, and Ly49P, which ligands are, respectively, H-2D^d^ and murine cytomegalovirus antigens. Two other members of this family, NK1.1 and NKR-P1F signals instead through FcεR1γ and recognize members of a new C-type lectin-related (Clr) family.

PILRβ has been identified as an activating NK cell receptor in mice, being its signaling dependent also on DAP12 and its ligand CD99.

Activating NK cell receptors used by mice and humans include CD16, CD244, the heterodimer CD94/NKG2C, the homodimer NKG2D, and DNAM-1.

Inhibitory NK cell receptors posses ITIM domains in their cytoplasmic tails, and its ligation results in activation of protein tyrosine phosphatases, mainly SHP-1 and SHP-2, that counteract the signals given by activating receptors, initiated by tyrosine phosphorylation.

Most of the killer cell immunoglobulin-like receptors (KIR) have inhibitory activity, being their ligands specific or broad HLA haplotypes. Among them, KIR2DL1 recognizes HLA-Cw4 and related, “group2” alleles. KIR2DL2 and KIR2DL3 recognize HLA-Cw3 and related, “group1” alleles. KIR3DL1 is the receptor for HLA-B allotypes with Bw4 motifs. Finally, KIR3DL2 is the receptor for HLA-A3/11 and KIR2DL5 ligands are unknown.

Some members of the C-lectin type family of NK cell receptors are inhibitory. These are the heterodimer CD94/NKG2A, which ligand is HLA-E, and the homodimers Mafa and NKR-P1 (CD161), which ligands, are, respectively, cadherins and lectin-like transcript 1 (LLT1), respectively.

Other human NK cell inhibitory receptors are immunoglobulin-like transcript 2 (ILT2), which binds to most HLA-I haplotypes; LAIR-1, which binds to collagen; CEACAM-1, that binds to the different CD66 variants; and SIGLEC7, that binds to sialic acids.

Finally, KIR2DL4 (CD158d) is a member of the KIR family, which ligand is the non-classical HLA protein HLA-G, that has both activating and inhibitory capacities, similar to the described situation with CD244 (2B4).

Again, most of the inhibitory receptors in mouse NK cells belong to the C-type lectin family, Ly49a, c, e, f, g, and i which ligands are different MHC-I haplotypes (see Table 1 for the specific ligands of each receptor). Other inhibitory receptors in mouse NK cells are gp49b1, that binds to integrins, and PILRα, which ligand is CD99. Inhibitory receptors shared by mouse and human NK cells are Mafa, CD94/NKG2A, and LAIR-1. See Table 1 for a summary of all receptors, ligands, and adaptors.

**Table 1:** **Natural killer cell activating and inhibitory receptors in mice and humans and their ligands**.

Adaptor	Activating receptor	Ligand	Inhibitory receptor	Ligand
**HUMAN AND MOUSE**				
FcεRIγ, CD3ζ	CD16	IgG	CD94-NKG2A	HLA-E (Qa1^b^)
	NKp46	HA, ?	Mafa	Cadherins
DAP12	CD94-NKG2C	HLA-E (Qa1^b^)	LAIR-1	Collagen
DAP10	NKG2D	MIC, ULBP (Rae-1, H60)		
SAP/EAT2	CD244 (2B4)*	CD48		
–	DNAM-1	CD112, CD155		
**HUMAN**				
FcεRIγ, CD3ζ	NKp30	B7-H6, ?	KIR2DL1	HLA-Cw4
			KIR2DL2	HLA-Cw3
			KIR2DL3	HLA-Cw3
DAP12	NKp44	?	KIR3DL1	HLA-Bw4
	KIR2DS1	HLA-Cw3	KIR3DL2	HLA-A3/11
	KIR2DS2	?	KIR2DL5	?
	KIR2DS4	HLA-Cw4	NKR-P1 (CD161)	LLT1
	KIR3DS1	?	ILT2	HLA-I
FcεRIγ	KIR2DL4*	HLA-G	CEACAM-1	CD66
SAP/EAT2	CRACC*	CRACC	SIGLEC7	Sialic acids
	NTB-A*	NTB-A		
–	NKp80	AICL		
**MOUSE**				
DAP12	Ly49D	H-2D^d^	Ly49a	H-2D^d^, -D^k^
	Ly49H	MCMVm157	Ly49c	H-2K^b^, -K^d^, -D^d^, -D^k^
	Ly49P	MCMV?		
	PILRβ	CD99	Ly49e	?
FcεRIγ	NK1.1	Clr?	Ly49f	H-2^d^
	NKR-P1F	Clrg	Ly49g	H-2D^d^
			Ly49i	H-2D^k^
			gp49b1	Integrins
			PILRα	CD99

The adaptors used by each family of activating receptors are also indicated. In the mouse and human activating receptor ligand row, parenthesis indicates the mouse ligand. *Indicates receptors with both activating and inhibitory capabilities.?, Unknown ligand.

## NK Cells in Tumor Immunotherapy

A large interest in NK cells is currently coming from the field of cancer immunotherapy, which tries to increase the anti-tumoral immune response of cancer patients. Data from several laboratories suggest that exploiting NK cell alloreactivity could be largely beneficial independently of the NK cell source for the treatment of blood-borne cancers (Terme et al., 2008; Velardi, 2008; Cho and Campana, 2009). Donor-versus-recipient NK cell reactivity is mediated by KIRs, which sense missing expression of donor KIR-ligand(s) in the recipient and mediate alloreactions. KIR-ligand mismatching is a prerequisite for NK cell alloreactivity because in 20 donor–recipient pairs that were not KIR-ligand mismatched in the graft-versus-host direction, no donor alloreactive NK clones were found (Ruggeri et al., 2007b).

The most advanced clinical use of NK cells is related to hematological cancers in which current clinical protocols fail inducing long-term survival in a significant number of patients. Those refractory to standard treatment, i.e., radio- and/or chemotherapy are often subjected to a myeloablative regimen followed by allogenic hematopoietic stem cell transplantation (HSCT). However, the mortality linked to this treatment approaches to 20%. In addition, Graft-versus-Host (GvH) and HvG diseases and opportunistic infections hamper this procedure. Moreover, relapse has become the leading cause of death following allogenic HSCT: the relapse rate has not decreased over the past 20 years (Kroger, 2011). In general, prognosis is poor for patients who relapsed to an allograft since effective treatment options are limited. These include donor lymphocyte infusions, withdrawal of immunosuppressive medication and second allogeneic HSCT. However, new specific cellular approaches are under investigation. In particular, the use of alloreactive NK cells after umbilical cord blood transplantation (UCBT) seems promising. KIR-ligand incompatibility in the GvH direction improves outcomes after UCBT in the clinic (Stern et al., 2008; Willemze et al., 2009). Moreover, NK cells: (1) are not responsible of GvHD; (2) can be injected as “differentiated” cells without the need of long time survival on patients body; (3) protect from opportunist infections (Willemze et al., 2009), probably through their immunoregulatory effects on B cells, T cells and macrophages, and more importantly on polymorphonuclear cells (PMNs; Bhatnagar et al., 2010). Finally, NK cell-mediated therapy after hematopoietic cell transplantation seems safe (Miller et al., 2005; Ruggeri et al., 2007a; Rubnitz et al., 2010). Immunotherapy, in particular NK cell-mediated, is probably the only approach to eliminate these highly resistant tumor cells.

*In vitro* studies on primary lympho-hematopoietic lineage tumor cells showed that alloreactive NK cells kill acute and chronic myeloid leukemia, as well as T cell acute lymphoblastic leukemia (ALL), chronic lymphocytic leukemia, non-Hodgkin’s lymphoma, and multiple myeloma. The only non-susceptible target was common ALL, however, KIR-ligand incompatibility in the GvH direction improves outcomes after UCBT in ALL patients. Despite their source, alloreactive NK cells home to lympho-hematopoietic sites and ablate recipient lympho-hematopoietic cells, including leukemic cells, while sparing other healthy organs. This explains why NK cells immunotherapy would be mainly useful in blood-borne cancers (Stern et al., 2008; Willemze et al., 2009).

New strategies are being developed to use NK cells in the treatment of solid tumors in the clinic. Several tumor-targeted monoclonal antibodies (mAbs) are included in the clinical care for certain tumors. Besides inducing antibody-dependent cell-mediated cytotoxicity (ADCC), these mAbs can kill their targets through activation of the complement, which in certain cases could be associated to clinical toxicity, i.e., anti-GD2 therapy (Sorkin et al., 2010). Nowadays the clinical use of mAbs tries to mainly exploit ADCC, which induces a more satisfactory clinical response and is mainly mediated by NK cells *in vivo* (Alderson and Sondel, 2011). Between the several activating or inhibitory Fc receptors for IgG (FcγR), NK cells express almost exclusively the activating FcγRIIIa (CD16). The importance of this receptor in the clinic is highlighted by the fact that patients with a valine at position 158 of FcγRIIIa (FcγRIIIa^158v^) respond better to mAbs-mediated therapy. This is linked to the higher affinity for IgG of FcγRIIIa^158v^ NK cells, leading to a more sensitivity activating receptor and increase ADCC (Alderson and Sondel, 2011).

## PKCθ in NK Cells

Compared to T cells, much less is known about the role of PKCθ in NK cells. PKCθ is expressed in NK cells (Balogh et al., 1999; Vyas et al., 2002b) and it has been demonstrated that it mediates the phosphorylation of WASP-interacting protein (WIP) during NK cell activation (Krzewski et al., 2006). In addition, it has been shown that PKCθ translocates to the IS during NK recognition of target cells (Davis et al., 1999).

It was shown that NK cells from PKCθ deficient mice had impaired IL-12-stimulated interferon (IFN)-γ production, without affecting their cytotoxic potential on YAC-1 cells (Page et al., 2008), which are extremely sensitive to NK cells, including naïve, *ex vivo* obtained, NK cells. However, in a different study (Tassi et al., 2008), no effect of PKCθ deficiency was observed on IFN-γ secretion induced by IL-12, IL-18, or a combination of both cytokines. In this study no effect of PKCθ deficiency was either observed on cytotoxicity against YAC-1 cells or against tumoral cells over-expressing KAR ligands (Tassi et al., 2008). It is also possible that the protocol used to generate NK cells, *ex vivo* culture in the presence of IL-2, may have masked the effect of PKCθ during the cellular cytotoxicity assays, since the tumor cells used in that study are especially sensitive to NK cells (Van den Broek et al., 1995; Screpanti et al., 2001; Pardo et al., 2002). Nevertheless, PKCθ was required to induce IFN-γ and TNF-α secretion by KAR that contain ITAMs such as NK1.1 and Ly49D. Finally, the early control of murine cytomegalovirus infection, that is dependent on NK cell activity, was not affected by the absence of PKCθ (Tassi et al., 2008).

In human γδ T cells the co-stimulation mediated by NKG2D was dependent on PKCθ (Nedellec et al., 2010). However, this study mostly relies on the use of the PKCθ inhibitor rottlerin, which has been shown to also inhibit other cellular kinases (Davies et al., 2000).

In addition, our group has demonstrated that PKCθ plays a prominent role in tumor immune surveillance mediated by NK cells (see below, Aguiló et al., 2009).

## PKCθ in Anti-Tumor Immune Surveillance

The cancer immuno-surveillance hypothesis proposes that the immune system detects and eliminates cells undergoing tumor transformation. Immuno-deficient mice develop more tumors than immuno-competent mice and clinical data support the notion that cancer immuno-surveillance also occurs in humans (Dunn et al., 2002, 2006; Aptsiauri et al., 2007). In addition, the adaptive immune system is thought to maintain small cancer lesions in an equilibrium state (Koebel et al., 2007; Melief, 2007). Therefore, the relevant cellular effectors of immuno-surveillance must perform two critical tasks to eradicate developing tumors: directly kill tumor cells and produce cytokines such as IFN-γ to stimulate the host immune response (Dunn et al., 2006).

We tested the role of PKCθ in T cell leukemia progression by inducing the disease in wild-type (*wt*) and PKCθ-deficient mice with moloney murine leukemia virus (M-MuLV). In line with the above-mentioned studies, we found that disease incidence and onset were enhanced in *PKC*θ^−/−^ mice. Transfer of leukemic T cells from *wt* donors into PKCθ-deficient and *wt* recipients induced leukemia in 100 and 40% of the mice, respectively. Interestingly, leukemic cells from *PKC*θ^−/−^ donors were less efficient at forming tumor since only 50% of the PKCθ-deficient and 10% of the *wt* recipients developed the disease. Consistent with these observations, intravenous injection of low numbers of the murine lymphoma T cell line EL4 induced tumors more rapidly in *PKC*θ^−/−^ mice compared to their wt counterpart. These results showed that PKCθ was essential for the immune response to leukemia in mice. This response probably involved CTL function, since both M-MuLV-induced tumors and EL4 cells expressed MHC-I (Garaude et al., 2008). These results also suggest a role of PKCθ in cancer immune surveillance, since tumors generated in the absence of this protein were less aggressive than those generated in the presence of PKCθ. In this connection, it has been also described that PKCθ, by mediating the activation of NF-κB by pre-TCR in immature thymocytes, contributes to the development of Notch3-dependent T cell lymphoma (Felli et al., 2005). This indicates that PKCθ could be somehow required for lymphoma cell “fitness,” and the results obtained in *PKC*θ^−/−^ mice could reflect both effects.

Cells of the innate immune system (γδ T, NK, and NK T cells) can also mediate anti-tumor responses. Specifically, NK cells play an important role in tumor immune surveillance (Ljunggren and Karre, 1985; Terme et al., 2008; Vivier et al., 2008), as they control progression of MHC-I-deficient tumors using perforin/granzyme- and FasL-mediated cytotoxicity (Van den Broek et al., 1995; Screpanti et al., 2001; Pardo et al., 2002). Moreover, NK cells also function as mediators between innate and adaptive immunity in anti-tumor responses (Moretta et al., 2008).

Since most tumors cells down-regulate MHC-I expression to escape the CTL-mediated response (Garrido et al., 2010), it is important to study the role of NK cells in this context.

The *in vivo* development of a MHC-I-deficient tumor (RMA-S) is favored in *PKC*θ^−/−^ mice compared with wild-type mice (Aguiló et al., 2009). Previous studies clearly demonstrated that the *in vivo* development of this tumor is not dependent on T cells and that it is controlled by NK cell activity (Kärre et al., 1986; Smyth et al., 1998, 2000; Kelly et al., 2002; Vosshenrich et al., 2005). The enhanced tumor growth in *PKC*θ^−/−^ mice was associated with a deficient recruitment of NK cells to the site of tumor development and with a decreased activation status of recruited NK cells. This correlated with a reduced *ex vivo* cytotoxic potential of NK cells isolated from *PKC*θ^−/−^ mice on RMA-S cells after poly I:C treatment (Aguiló et al., 2009). Interestingly, and confirming previous data (Page et al., 2008; Tassi et al., 2008), no difference was observed on the killing of YAC-1 cells suggesting that in addition to the absence of MHC-I, other regulatory events might be required for the tight control of NK cytotoxicity. YAC-1 cells are sensitive to naïve NK cells and are not able to induce tumors in syngeneic mice because of their extreme sensitivity to NK cell-mediated lysis, mediated by both perforin/granzymes and FasL (Aguiló et al., 2009). However, NK cells do not target RMA-S cells unless they are previously activated, i.e., by *in vivo* injection of poly I:C. Interestingly, only perforin/granzymes, but not FasL, are responsible for the elimination of RMA-S cells mediated by activated NK cells (Pardo et al., 2002; Aguiló et al., 2009). Adoptive transfer of naïve NK cells from wt or *PKC*θ^−/−^ mice to *PKC*θ^−/−^ mice was also performed to demonstrate that the defect in activation was intrinsic to NK cells, and not due to any other cellular component. Hence, PKCθ seems to be implicated in NK cell-mediated anti-tumor immunity at least by acting on the cytolytic potential of activated NK cells.

Poly I:C has been extensively used as an indirect *in vivo* NK cell activator, through the secretion of cytokines by macrophages and/or dendritic cells (Djeu et al., 1979). Poly I:C, mimics viral double-stranded RNA, and is recognized by the member of the Toll-like receptor family TLR3, which is expressed by antigen-presenting cells (Alexopoulou et al., 2001), and also by cytosolic receptors such as MDA5 and RIG-I (Kato et al., 2006). Poly I:C treatment increased the level of expression and the activation status of PKCθ in NK cells, both *in vivo* and *in vitro*. In the latter case, the presence of the whole splenocyte population was needed, being the effect presumably mediated by macrophages and/or dendritic cells (Aguiló et al., 2009). This was in agreement with previous studies demonstrating the increase in anti-tumor activity of NK cells activated by poly I:C (Akazawa et al., 2007). The increase in PKCθ expression depended on cell-to-cell contact, while its activation was mediated by a soluble factor. This soluble factor should be one of the cytokines secreted by macrophages and/or DCs that are known to activate NK cells. Since IL-12 signal transduction was reported to be affected in NK cells from *PKC*θ^−/−^ mice (Page et al., 2008), this cytokine was tested first. IL-15 was also included in those studies, since it is important in regulating NK cell function and survival (Carson et al., 1994; Cooper et al., 2002), and for efficient anti-tumoral NK cell activity (Liu et al., 2012). Indeed, both, IL-12 and IL-15, activated PKCθ in NK cells, with IL-15 being more potent at inducing PKCθ phosphorylation. More importantly, neutralizing antibodies to IL-15, but not those blocking IL-12, reduced substantially NK cell PKCθ phosphorylation induced *in vitro* by poly I:C treatment of a mixed splenocyte population (Aguiló et al., 2009). How could IL-15 be coupled to PKCθ activation? Interestingly, PKCθ is the only T cell expressed PKC isoform that is activated through a PI3K-dependent pathway, which is also activated by TCR ligation (Villalba et al., 2002). Although the main signaling pathway elicited by cytokine receptors is mediated by JAK and STATs, cytokines such as IL-2 are also able to activate the PI3K pathway (Brennan et al., 1997). It is interesting to note that IL-2 and IL-15 share the same signaling receptors suggesting that IL-15 could also trigger the PI3K pathway, as has been suggested in some studies in T cells (Ben Ahmed et al., 2009).

Therefore, IL-15 looked as a very feasible candidate to be a mediator in PKCθ-dependent anti-tumoral NK cell activation. We have performed additional experiments, analyzing the effect of IL-15 on different NK cell functions. We have found that, although IL-15 is able to signal through PKCθ, this signaling is not needed for the main functional effects of IL-15 on NK cells (Aguiló et al., in preparation). Hence, IL-15 probably is not the main cytokine implicated in PKCθ-dependent NK cell anti-tumoral activity. Additional experiments will be required to uncover cytokines that mediate dendritic cell-mediated NK cell activation.

**Figure 2:**
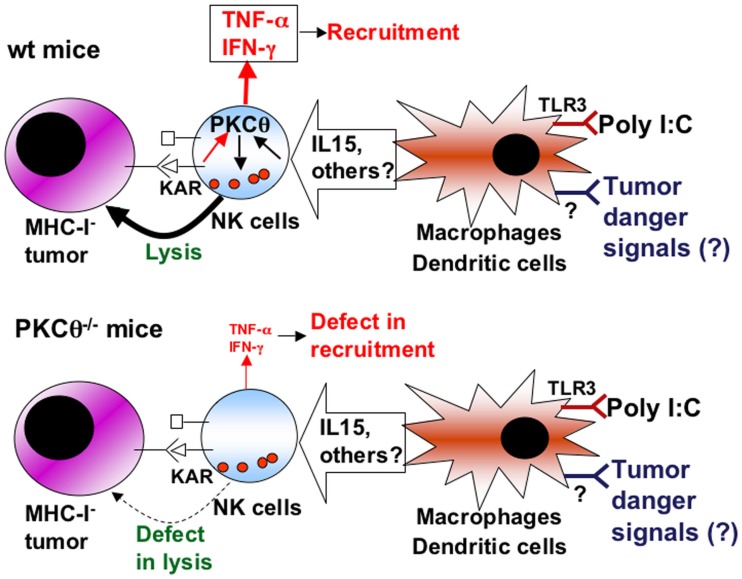
**Schematic showing the role of PKCθ in anti-tumoral NK cell activation**. Upper panel, situation in NK cells from wild-type (wt) mice: cytokines produced by macrophages and/or dendritic cells upon poly I:C activation, or upon the sensing of tumor “danger” signals, including IL-15, induce the activation of NK cells through signaling dependent on PKC-θ (black arrows). This activation increases the cytotoxic potential of NK cells that can lyse tumoral target cells, especially those that are negative for MHC-I expression, and prevent tumor development. In addition, the production of TNF-α and IFN-γ by NK cells induced by certain killer-activating receptors (KAR) is also dependent on PKC-θ (red arrows), allowing the recruitment of more immune cells and sustaining immune activation. Lower panel, situation in NK cells from PKC-θ^−/−^ mice: as a consequence of the absence of PKC-θ, cytokines produced by macrophages and/or dendritic cells do not activate properly NK cells, which then show a defect in lysis of tumoral cells, allowing tumoral development. In addition, the production of TNF-α and IFN-γ by NK cells is decreased, causing defects in recruitment.

As a summary (Figure 2), we propose that poly I:C, through TLR3 activation on macrophages or dendritic cells, elicits the secretion of cytokines that activate NK cells, including IL-15. During tumor development, danger signals are generated that activate antigen-presenting cells, which in turn ensure tumor antigen presentation and secrete a set of cytokines that regulate NK cells. Some of these cytokines may signal through PKCθ to control NK cell activation. This activated state is needed to counteract the growing of MHC-I negative tumors such as RMA-S, while other tumors, extremely sensitive to NK cell-mediated cytotoxicity, such as YAC-1, do not grow in syngenic mice. Once KARs are activated through the ligands expressed on tumoral cells, activated NK cells would be able to eliminate the tumor in a defined cytokine environment. On the other hand, KAR ligation induces the NK cell secretion of TNF-α and IFN-γ from activated NK cells, a process in which PKCθ is implicated (Tassi et al., 2008). TNF-α is important for the recruitment of more NK cells to the tumor environment (Smyth et al., 1998), which could explain the reduced recruitment of NK cells observed in *PKC*θ^−/−^ mice (Aguiló et al., 2009).

## A Role for PKCθ in NK Cell Degranulation?

Natural killer cells from *PKC*θ^−/−^ mice had a reduced capacity to degranulate against RMA-S cells, which correlated with the impairment in their lytic ability (Aguiló et al., 2009). It has been recently shown that PKC-θ, together with other members of the novel PKC family, is implicated in the formation of the IS in CD4^+^ T cells, probably through a function in microtubule-organizing center (MTOC) polarization (Quann et al., 2011). However, this study did not address the possible involvement of this mechanism in lytic granule secretion and on cytotoxicity.

### Immunological synapse formation

Most of the studies performed to determine the relevant molecular components in lytic granule secretion have been realized on CTL. The IS of CTL is quite distinct from that of CD4^+^ T cells since it should allow both signaling and degranulation. The description of the CTL immune synapse showed the presence of a “cleft” that allowed the secretion of lytic granules components (Stinchcombe et al., 2001b). Hence, the signaling regulating synapse formation could be different in CD4^+^ and CD8^+^ T cells, and even if this signaling is shared, the regulation of lytic granule secretion should be particular to cytotoxic cells (CTL and NK cells). At the functional level, not all cytokines are secreted into the synapse of CD4^+^ T cells: IL-2 and IFN-γ follow a synapse-mediated secretion, while TNF-α and some chemokines do not follow a directional type of secretion (Huse et al., 2006). On the other hand, cytokine release and degranulation proceed by different pathways also in NK cells (Reefman et al., 2010).

The IS of NK cells is similar to the one described for CTLs although it has some particularities (Davis et al., 1999; Krzewski and Strominger, 2008). Two different IS have been named, the activating IS and the inhibitory IS. Activating IS is formed by a central supramolecular activating cluster (SMAC) where all receptors involved in signal transduction accumulate and a peripheral SMAC where the adhesion molecule LFA-1 is placed. Similarly, inhibitory receptors accumulate in the cSMIC and LFA-1 in the pSMIC (Carlin et al., 2001). However, given the different number of activating and inhibitory receptors (see Natural Killer Cells), the molecular mechanisms regulating signaling from the synapse is more complex than in the case of other lymphocytes subsets like CTLs (Vyas et al., 2002a; Krzewski and Strominger, 2008). Specifically, formation and signaling through IS in NK cells is dynamically regulated by activation thresholds dictated by the balance between activating and inhibitory receptors. Most of the studies have characterized the IS formed after engagement of target cells by the activating NKG2D receptor (Krzewski and Strominger, 2008). However, as explained above, signaling from NKG2D receptors is regulated differentially from other receptors. Formation of the NK cell IS is regulated at different stages (Orange et al., 2003). The first stage implies an activation process after target cell engagement, followed by signal amplification and the rearrangement of the actin cytoskeleton in the pSMAC. This process is regulated by the WASP protein. Subsequently, MTOC is polarized and granule content is released through the cSMAC, as in the case of CTLs. All these activation steps are negatively regulated by signaling transduced by SHP phosphatases from inhibitory receptors (Krzewski and Strominger, 2008).

### Molecules regulating CTL and NK cell degranulation

The molecular defects responsible of several diseases have allowed the identification of important molecules implicated in CTL degranulation (Clark and Griffiths, 2003; Stinchcombe and Griffiths, 2007; de Saint Basille et al., 2010). Granule movement through tubulin filaments is regulated by the adaptor protein-4 (AP-3; Clark et al., 2003), docking onto the plasma membrane is regulated by Rab27a (Haddad et al., 2001; Stinchcombe et al., 2001a), and priming or fusion with the membrane by Munc-13-4 (Feldmann et al., 2003). Finally, ASMAse is involved in the proper shrinkage of secretory granules after docking and priming steps to efficiently release granule content into target cells (Herz et al., 2009).

Regarding NK cells, it has been demonstrated that myosin IIA and WIP are required for granule polarization and NK cell-mediated cytotoxicity (Krzewski et al., 2006). Moreover, it could be suggested that Munc-13-4 is also involved in NK cell granule exocytosis since patients deficient in this protein present deficient NK cell-mediated cytotoxicity (Marcenaro et al., 2006; Bryceson et al., 2007). Syntaxin-11 also participates in the fusion of granules with the membrane of human NK cells (Arneson et al., 2007; Bryceson et al., 2007).

### TCR early signaling during CTL degranulation. Implication of PKC isoforms

Although the most important regulators of lytic granule exocytosis are well described, at least in CTL, the connection between activating receptor-triggered signal transduction and lytic granule secretion is not completely understood. In the case of CTL, TCR signal transduction is initiated through protein tyrosine kinases of the src family that, together with the action of recruited ZAP-70 results in the phosphorylation and activation of phospholipase C-γ1 (Mustelin et al., 1990; Chan et al., 1992). CTL degranulation depends on these tyrosine kinase-mediated signaling pathways (Secrist et al., 1991; O’Rourke and Mescher, 1992; Anel and Kleinfeld, 1993). PLC-γ activation produces diacyl glycerol (DAG), which activates PKCs, and inositol-trisphosphate (IP_3_), which increases the intracellular Ca^++^ concentration. A combination of the phorbol ester phorbol myristate-acetate (PMA) and the calcium ionophore ionomycin induces CTL degranulation. Granule movement was dependent on Ca^++^ and CTL shape change was dependent on PKC activity (Takayama and Sitkovsky, 1987; Haverstick et al., 1991). Depletion of PMA-sensitive PKC isoforms by prolonged PMA exposure prevented TCR-induced degranulation in CTL clones (Nishimura et al., 1987; Anel et al., 1994). In addition, PKC inhibition prevented MTOC polarization in CTL (Nesic et al., 1998).

A closer look at the role of individual PKC isoforms in CTL function revealed that PKCθ was implicated in the induction of FasL expression at a transcriptional level (Villalba et al., 1999; Villunger et al., 1999; Pardo et al., 2003). This is probably due to the role of PKCθ in NF-AT and NF-κB activation (Sun et al., 2000; Pfeifhofer et al., 2003), transcription factors which are involved in the control of FasL gene transcription (Latinis et al., 1997; Kasibhlatla et al., 1999). As already commented above, PKCθ is the only PKC isoform that is activated through a PI3K-dependent pathway that is triggered by TCR ligation (Villalba et al., 2002). This is in agreement with the fact that functional FasL expression is also prevented by PI3K inhibitors in long-term CTL clones (Anel et al., 1995; Pardo et al., 2003). Perforin/granzyme mediated lysis of Fas-negative target cells and CTL degranulation is sensitive to broad-spectrum PKC inhibitors, and also to Gö-6976, a specific inhibitor of the classical PKC isoforms (α, β, γ), but not to low doses of rottlerin, an inhibitor that prevents PKCθ activation (Villalba et al., 1999; Pardo et al., 2003). In addition, transfection with constitutively active PKCα, but not with PKCθ, cooperated with ionomycin to promote degranulation in murine CTL clones (Pardo et al., 2003). Later works demonstrated that both constitutively active PKCα and PKCθ cooperated with thapsigargin to induce degranulation in a human CTL tumoral cell line, although PKCα seemed more efficient (Grybko et al., 2007).

### Downstream signaling during CTL degranulation

Cytotoxic T lymphocyte and NK cell degranulation is dependent on both actin cytoskeleton and on tubulin microtubules (Gomez and Billadeau, 2008). The proximal signaling described above connects initially with remodeling of the actin cytoskeleton mainly through the adaptors Vav1 and SLP76 (Villalba et al., 2001a; Zeng et al., 2003). These actin cytoskeleton remodeling events are needed for immune synapse formation in CD4^+^ T cells, in CTL and also in NK cells. Once the immune synapse is formed, the next step for degranulation to occur is MTOC polarization (Kuhn and Poenie, 2002). Once MTOC is polarized, granules should move on tubulin rails, dock to the plasma membrane, and fuse to release their content in the IS. For these steps to occur, the granule and membrane proteins described above are needed, but signaling events are not completely elucidated. A role for ERK activation in CTL degranulation was clearly established (Berg et al., 1998). This activation was dependent on PI3K, and paxillin has been identified as the ERK substrate that mediates MTOC polarization to the IS (Robertson et al., 2005; Robertson and Ostergaard, 2011). However, a role for PKCθ in ERK activation was not clearly demonstrated, and primary CTL from *PKC*θ^−/−^ mice degranulated normally (Puente et al., 2006).

### Early signaling during NK cell degranulation

Regarding NK cell degranulation elicited by KARs, signal transduction pathways are similar to those described in CTL in the case of those receptors that use FcεR1γ, CD3ζ, or DAP12 adaptors, but different in receptors that use DAP10 or SAP adaptors, such as NKG2D and CD244, respectively. However, by activating the PI3K pathway and the PLCγ-mediated increase in intracellular calcium concentration in the case of NKG2D and by the SAP-mediated activation of the protein tyrosine kinase Fyn in the case of CD244, these KAR arrive to generate similar downstream signaling that leads finally to degranulation (Cerwenka and Lanier, 2001; Lanier, 2008). In fact, it has been demonstrated that mouse NK cells do not require Syk and ZAP-70 kinases to kill different types of target cells, even those that do not express ligands for NKG2D, and that only abrogating at the same time the activity of src kinases and PI3K, degranulation was prevented (Colucci et al., 2002). In addition, the consequences of signaling transduced by the different receptors do not seem to be redundant. It has been previously shown that not a single activating receptor is sufficient to induce NK cell-mediated cytotoxicity against target cells (Bryceson et al., 2006). Only certain synergistic combinations of receptors are able to trigger this process. For example, it has been shown that engagement of CD16 induces degranulation in a non-polarized manner, meanwhile engagement of LFA-1 was able to polarize granules toward the IS. However, only after simultaneous engagement of both molecules NK cells were able to kill target cells (Bryceson et al., 2005), indicating also the importance of adhesión receptors such as LFA-1.

### Downstream signaling during NK cell degranulation. Implication of PKC

Regarding downstream signaling in NK cell degranulation, it has been clearly demonstrated that the PI3K-mediated activation of ERK is involved in NK cell degranulation elicited by CD16 (Bonnema et al., 1994), by KARs that use DAP12 (Jiang et al., 2000, 2002) and also by those using DAP10 as adaptor (Billadeau et al., 2003; Upshaw et al., 2006). ERK activation results finally in MTOC polarization, similarly to what was observed in CTL degranulation (Chen et al., 2006). In the case of NKG2D, the PI3K pathway also results in the recruitment of the adaptor CrkL, needed for efficient MTOC polarization (Segovis et al., 2009). A role for JNK activation has been also demonstrated in NKG2D-mediated cytotoxicity (Li et al., 2008).

The role of PKC in NK cell degranulation was initially demonstrated since a combination of PMA and ionomycin is able to induce degranulation in these cells (Bonnema et al., 1994). It was also demonstrated that DNAM-1 signal transduction is dependent on PKC expression (Shibuya et al., 1998). Less is known about the role of different PKC isoforms in NK cell degranulation. As mentioned above, PKCθ is expressed by NK cells. During activation of the human NK cell line YTS with target cells with no HLA-I expression, a PKCθ pseudo-substrate inhibitor prevented WIP phosphorylation (Krzewski et al., 2006). WIP forms a complex with WASP and myosin IIA that regulates actin cytoskeleton dynamics during NK cell activation, and WIP knockdown prevents NK cell degranulation (Krzewski et al., 2008). However, it was not demonstrated that the formation of this complex was dependent on WIP phosphorylation by PKCθ (Krzewski et al., 2006). Interestingly, it has been recently published the kinome of NK cells activated by CD16 or simultaneous CD244 and DNAM-1 ligation, being PKC-θ identified as the only PKC isoform that increase phosphorylation upon receptor ligation (König et al., 2012).

The results described above, most of them obtained in CTL, suggest that PKCθ is not mandatory for granule exocytosis. However, it has not been studied yet in detail its possible involvement in NK cell immune synapse signaling and in NK cell granule polarization and/or secretion.

## Conclusion

PKCθ plays an important role in tumor immune surveillance *in vivo* (Garaude et al., 2008; Aguiló et al., 2009). In the case of CTL-mediated tumor control, this can be explained by its role as a CTL survival factor (Barouch-Bentov et al., 2005), the impaired cytokine response observed in PKCθ deficient animals (Sun et al., 2000) and its implication in FasL expression (Villalba et al., 1999; Villunger et al., 1999; Pardo et al., 2003).

**Figure 3:**
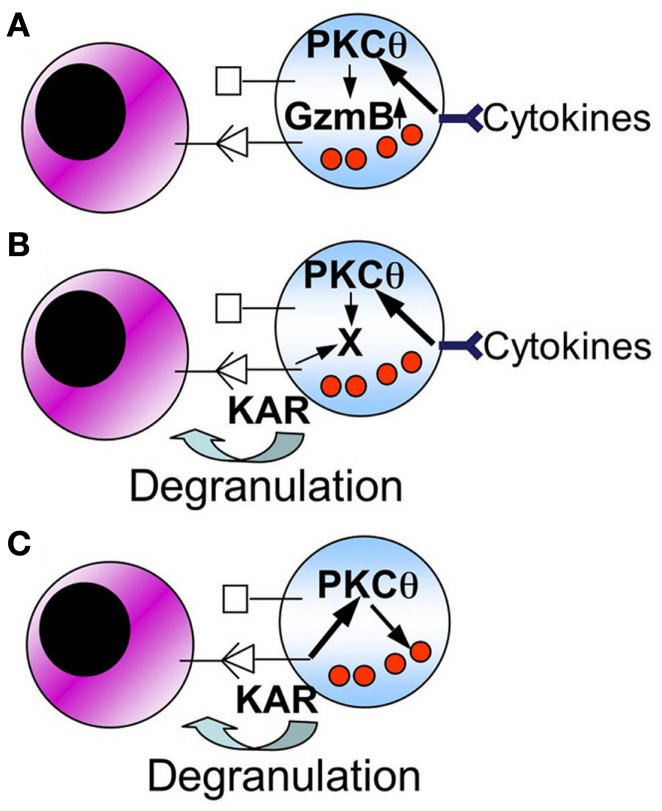
**Schematic showing possible mechanisms for the role of PKCθ in NK cell-mediated anti-tumor immunity**. The following working hypothesis are formulated (see the text for details): **(A)** cytokines produced by macrophages and/or dendritic cells, by signaling through PKC-θ, increase granzyme B expression. An increase in granzyme B expression is directly associated with the augmentation of the cytolytic potential of NK cells; **(B)** cytokines produced by macrophages and/or dendritic cells, by signaling through PKC-θ, induce or increase the expression of a protein (X) implicated in degranulation of NK cells; **(C)** PKC-θ could be directly implicated in degranulation of NK cells through specific KARs.

In the case of NK cell-mediated tumor control, several functional consequences of PKCθ deficiency can also contribute to the final outcome. As depicted in the schematic Figure 2, KAR-induced TNF-α and IFN-γ secretion is defective in *PKC*θ^−/−^ mice (Tassi et al., 2008), and this can contribute to a defective recruitment of effector cells to the site of tumor development (Aguiló et al., 2009). Also, PKCθ could be implicated in the induction of FasL expression (Pardo et al., 2003), although this has not been demonstrated in NK cells. In addition, the possibilities shown in Figure 3 may be considered, although they should be taken as working hypothesis that need to be better characterized:

a) Macrophage or dendritic cell derived cytokines, through a pathway that implicates PKCθ, induce an increase in granzyme B expression.
b) Macrophage or dendritic cell derived cytokines, through a pathway that implicates PKCθ, induces or increases the expression of a cellular component (X) that is needed to increase the degranulation potential of NK cells.
c) PKCθ is directly implicated in NK cell degranulation elicited by specific KARs.

These three possibilities are not mutually exclusive, and all of them can contribute to the regulation of NK cell anti-tumoral potential. In addition, different signaling and/or functional responses can be activated by different KARs, and PKC-θ could be relevant for signaling elicited by some KARs but not by others. Undoubtedly, uncovering the precise molecular mechanisms that direct these processes will allow a rational improvement of NK cell-mediated anti-tumor immunotherapy protocols.

## Conflict of Interest Statement

The authors declare that the research was conducted in the absence of any commercial or financial relationships that could be construed as a potential conflict of interest.

## References

[B1] Aguiló J. I. Garaude J. Pardo J. Villalba M. Anel A. (2009). Protein kinase C-theta is required for NK cell activation and *in vivo* control of tumor progression. J. Immunol. 182, 1972–1981 10.4049/jimmunol.0801820 19201850

[B2] Akazawa T. Ebihara T. Okuno M. Okuda Y. Shingai M. Tsujimura K. Takahashi T. Ikawa M. Okabe M. Inoue N. Okamoto-Tanaka M. Ishizaki H. Miyoshi J. Matsumoto M. Seya T. (2007). Antitumor NK activation induced by the Toll-like receptor 3-TICAM-1 (TRIF) pathway in myeloid dendritic cells. Proc. Natl. Acad. Sci. U.S.A. 104, 252–257 10.1073/pnas.0605978104 17190817 PMC1765444

[B3] Alderson K. L. Sondel P. M. (2011). Clinical cancer therapy by NK cells via antibody-dependent cell-mediated cytotoxicity. J. Biomed. Biotechnol. 2011, 379123 10.1155/2011/379123 21660134 PMC3110303

[B4] Alexopoulou L. Holt A. C. Medzhitov R. Flavell R. A. (2001). Recognition of double-stranded RNA and activation of NF-κappaB by Toll-like receptor 3. Nature 413, 732–738 10.1038/35099560 11607032

[B5] Altman A. Kaminski S. Busuttil V. Droin N. Hu J. Tadevosyan Y. Hipskind R. A. Villalba M. (2004). Positive feedback regulation of PLCγ1/Ca2^+^ signaling by PKCθ in restimulated T cells via a Tec kinase-dependent pathway. Eur. J. Immunol. 34, 2001–2011 10.1002/eji.200324625 15214048

[B6] Anel A. Kleinfeld A. M. (1993). Tyrosine phosphorylation of a 100 kDa protein is correlated with cytotoxic T-lymphocyte function. Evidence from cis unsaturated fatty acid and phenylarsineoxide inhibition. J. Biol. Chem. 268, 17578–17587 8394347

[B7] Anel A. Richieri G. V. Kleinfeld A. M. (1994). A tyrosine phosphorylation requirement for cytotoxic T-lymphocyte degranulation. J. Biol. Chem. 269, 9506–9513 7511589

[B8] Anel A. Simon A. K. Auphan N. Buferne M. Boyer C. Golstein P. Schmitt-Verhulst A. M. (1995). Two signalling pathways can lead to Fas ligand expression in CD8+ cytotoxic T lymphocyte clones. Eur. J. Immunol. 25, 3381–3387 10.1002/eji.1830251227 8566027

[B9] Aptsiauri N. Cabrera A. García-Lora A. López-Nevoti M. A. Ruiz-Cabello F. Garrido F. (2007). MHC class I antigens and immune surveillance in transformed cells. Int. Rev. Cytol. 256, 139–189 10.1016/S0074-7696(07)56005-5 17241907

[B10] Arneson L. Brickshawana A. Segovis C. Schoon R. Dick C. Leibson P. J. (2007). Syntaxin 11 regulates lymphocyte-mediated secretion and cytotoxicity. J. Immunol. 179, 3397–3401 17785771 10.4049/jimmunol.179.6.3397

[B11] Baier G. Telford D. Giampa K. M. Coggeshall K. M. Baier-Bitterlich G. Isakov N. Altman A. (1993). Molecular cloning and characterization of PKCθ, a human novel member of the protein kinase C (PKC) gene family expressed predominantly in hematopoietic cells. J. Biol. Chem. 268, 4997–5004 8444877

[B12] Baier-Bitterlich G. Uberall F. Bauer B. Fresser F. Wachter H. Grunicke H. Utermann G. Altman A. Baier G. (1996). Protein kinase C-θ isoenzyme selective stimulation of the transcription factor complex AP-1 in T lymphocytes. Mol. Cell. Biol. 16, 1842–1850 8657160 10.1128/mcb.16.4.1842 PMC231171

[B13] Balogh G. de Boland A. R. Boland R. Barja P. (1999). Effect of 1,25[OH](2)-vitamin D(3) on the activation of natural killer cells: role of protein kinase C and extracellular calcium. Exp. Mol. Pathol. 67, 63–74 10.1006/exmp.1999.2264 10527758

[B14] Barouch-Bentov R. Lemmens E. E. Hu J. Janssen E. M. Droin N. M. Song J. Schoenberger S. P. Altman A. (2005). Protein kinase C-θ is an early survival factor required for differentiation of effector CD8^+^ T cells. J. Immunol. 175, 5126–5134 16210616 10.4049/jimmunol.175.8.5126

[B15] Ben Ahmed M. Belhadj Hmida N. Moes N. Buyse S. Abledadhim M. Louzir H. Cerf-Bensussan N. (2009). IL-15 renders conventional lymphocytes resistant to suppressive functions of regulatory T cells through activation of the phosphatidylinositol 3-kinase pathway. J. Immunol. 182, 6763–6770 10.4049/jimmunol.0801792 19454671

[B16] Berg N. N. Puente L. G. Dawicki W. Ostergaard H. L. (1998). Sustained TCR signaling is required for MAPK activation and degranulation by CTL. J. Immunol. 161, 2919–2924 9743353

[B17] Bertolotto C. Maulon L. Filippa N. Baier G. Auberger P. (2000). Protein kinase C theta and epsilon promote T-cell survival by a rsk-dependent phosphorylation and inactivation of BAD. J. Biol. Chem. 275, 37246–37250 10.1074/jbc.M007732200 10976111

[B18] Bhatnagar N. Hong H. S. Krishnaswamy J. K. Haghikia A. Behrens G. M. Schmidt R. E. Jacobs R. (2010). Cytokine-activated NK cells inhibit PMN apoptosis and preserve their functional capacity. Blood 116, 1308–1316 10.1182/blood-2010-01-264903 20501895

[B19] Billadeau D. D. Upshaw J. L. Schoon R. A. Dick C. J. Leibson P. J. (2003). NKG2D-DAP10 triggers human NK cell-mediated killing via a Syk-independent regulatory pathway. Nat. Immunol. 4, 557–564 10.1038/ni929 12740575

[B20] Bonnema J. D. Karnitz L. M. Schoon R. A. Abraham R. T. Leibson P. J. (1994). Fc receptor stimulation of phosphatidylinositol 3-kinase in natural killer cells is associated with protein kinase C-independent granule release and cell-mediated cytotoxicity. J. Exp. Med. 180, 1427–1435 10.1084/jem.180.4.1427 7931075 PMC2191702

[B21] Brennan P. Babage J. W. Burgering B. M. T. Gromer B. Reif K. Cantrell D. A. (1997). Phosphatidylinositol 3-kinase couples the interleukin-2 receptor to the cell cycle regulator E2F. Immunity 7, 679–689 10.1016/S1074-7613(00)80388-X 9390691

[B22] Bryceson Y. March M. Ljunggren H. Long E. (2006). Synergy among receptors on resting NK cells for the activation of natural cytotoxicity and cytokine secretion. Blood 107, 159–166 10.1182/blood-2005-04-1351 16150947 PMC1895346

[B23] Bryceson Y. Rudd E. Zheng C. Edner J. Ma D. Wood S. Bechensteen A. Boelens J. J. Celkan T. Farah R. A. Hultenby K. Winiarski J. Roche P. A. Nordenskjöld M. Henter J. I. Long E. O. Ljunggren H. G. (2007). Defective cytotoxic lymphocyte degranulation in syntaxin-11 deficient familial hemophagocytic lymphohistiocytosis 4 (FHL4) patients. Blood 110, 1906–1915 10.1182/blood-2007-02-074468 17525286 PMC1976360

[B24] Bryceson Y. T. March M. Barber D. F. Ljunggren H. G. Long E. O. (2005). Cytolytic granule polarization and degranulation controlled by different receptors in resting NK cells. J. Exp. Med. 202, 1001–1012 10.1084/jem.20051143 16203869 PMC2213171

[B25] Carlin L. Eleme K. McCann F. Davis D. (2001). Intercellular transfer and supramolecular organization of human leukocyte antigen C at inhibitory natural killer cell immune synapses. J. Exp. Med. 194, 1507–1517 10.1084/jem.194.10.1507 11714757 PMC2193674

[B26] Carson W. E. Giri J. G. Lindemann M. J. Linett M. L. Ahdieh M. Paxton R. Anderson D. Eisenmann J. Grabstein K. Caligiuri M. A. (1994). Interleukin (IL) 15 is a novel cytokine that activates human natural killer cells via components of the IL-2 receptor. J. Exp. Med. 180, 1395–1403 10.1084/jem.180.4.1395 7523571 PMC2191697

[B27] Cerwenka A. Lanier L. L. (2001). Natural killer cells, viruses and cancer. Nat. Rev. Immunol. 1, 41–49 10.1038/35095564 11905813

[B28] Chan A. C. Iwashima M. Turck C. W. Weiss A. (1992). ZAP-70: a 70 kd protein-tyrosine kinase that associates with the TCR ζ chain. Cell 71, 649 10.1016/0092-8674(92)90598-7 1423621

[B29] Chen X. Allan D. S. Krzewski K. Ge B. Koopcow H. Strominger J. L. (2006). CD28-stimulated ERK2 phosphorylation is required for polarization of the microtubule organizing center and granules in YTS NK cells. Proc. Natl. Acad. Sci. U.S.A. 103, 10346–10351 10.1073/pnas.0606381103 16801532 PMC1502460

[B30] Cho D. Campana D. (2009). Expansion and activation of natural killer cells for cancer immunotherapy. Korean J. Lab. Med. 29, 89–96 10.3343/kjlm.2009.29.6.589 19411773 PMC2771620

[B31] Clark R. Griffiths G. M. (2003). Lytic granules, secretory lysosomes and disease. Curr. Opin. Immunol. 15, 516–521 10.1016/S0952-7915(03)00113-4 14499259

[B32] Clark R. H. Stinchcombe J. C. Day A. Blott E. Booth S. Bossi G. Hamblin T. Davies E. G. Griffiths G. M. (2003). AP3-dependent microtubule-mediated movement of lytic granules to the immunological synapse. Nat. Immunol. 4, 1111–1120 10.1038/ni1000 14566336

[B33] Colucci F. Schweighoffer E. Tomasello E. Turner M. Ortaldo J. Vivier E. Tybulewicz V. Di Santo J. (2002). Natural cytotoxicity uncoupled from the Syk and ZAP-70 intracellular kinases. Nat. Immunol. 3, 288–294 10.1038/ni764 11836527

[B34] Cooper M. A. Fehniger T. A. VanDeusen J. B. Waite R. E. Liu Y. Aguila H. L. Caligiuri M. A. (2002). *In vivo* evidence for a dependence on interleukin 15 for survival of natural killer cells. Blood 100, 3633–3638 12393617 10.1182/blood-2001-12-0293

[B35] Coudronniere N. Villalba M. Englund N. Altman A. (2000). NF-kB activation induced by TCR/CD28 costimulation is mediated by PKC-θ. Proc. Natl. Acad. Sci. U.S.A. 97, 3394–3399 10.1073/pnas.060028097 10716728 PMC16250

[B36] Davies S. P. Reddy H. Caivano M. Cohen P. (2000). Specificity and mechanism of action of some commonly used protein kinase inhibitors. Biochem. J. 351, 95–105 10.1042/0264-6021:3510095 10998351 PMC1221339

[B37] Davis D. Chiu I. Fassett M. Cohen G. Mandelboim O. Strominger J. (1999). The human natural killer cell immune synapse. Proc. Natl. Acad. Sci. U.S.A. 96, 15062–15067 10.1073/pnas.96.11.6541 10611338 PMC24773

[B38] de Saint Basille G. Ménasché G. Fischer A. (2010). Molecular mechanisms of biogenesis and exocytosis of cytotoxic granules. Nat. Rev. Immunol. 11, 568–579 10.1038/nri2803 20634814

[B39] Dennehy K. M. Klimosch S. N. Steinle A. (2011). NKp80 uses an atypical hemi-ITAM to trigger NK cytotoxicity. J. Immunol. 186, 657–661 10.4049/jimmunol.0904117 21149606

[B40] Djeu J. Y. Heinbaugh J. A. Holden H. T. Herberman R. B. (1979). Role of macrophages in the augmentation of mouse NK cell activity by Poly I:C and interferon. J. Immunol. 122, 182–188 216746

[B41] Dunn G. P. Bruce A. T. Ikeda H. Old L. J. Schreiber R. D. (2002). Cancer immunoediting: from immunosurveillance to tumor escape. Nat. Immunol. 3, 991–998 10.1038/ni1102-991 12407406

[B42] Dunn G. P. Koebel C. M. Schreiber R. D. (2006). Interferons, immunity and cancer immunoediting. Nat. Rev. Immunol. 6, 836–848 10.1038/nri1961 17063185

[B43] Feldmann J. Callebaut I. Raposo G. Certain S. Bacq D. Dumont C. Lambert N. Ouachee-Chardin M. Chedeville G. Tamary H. Minard-Colin V. Vilmer E. Blanche S. Le Deist F. Fischer A. de Saint Basile G. (2003). Munc13-4 is essential for cytolytic granules fusion and is mutated in a form of familial hemophagocytic lymphohistiocytosis (FHL3). Cell 115, 461–473 10.1016/S0092-8674(03)00855-9 14622600

[B44] Felli M. Vacca A. Calce A. Bellavia D. Campese A. Grillo R. Di Giovine M. Checquolo S. Talora C. Palermo R. Di Mario G. Frati L. Gulino A. Screpanti I. (2005). PKC theta mediates pre-TCR signaling and contributes to Notch3-induced T-cell leukemia. Oncogene 24, 992–1000 10.1038/sj.onc.1208302 15592506

[B45] Garaude J. Kaminski S. Cherni S. Aguiló J. I. Jacquet C. Plays M. Rodriguez F. Hernández J. Hipskind R. A. Anel A. Villalba M. (2008). Impaired anti-leukemic immune response in PKCθ-deficient mice. Mol. Immunol. 45, 3463–3469 10.1016/j.molimm.2008.03.016 18462800

[B46] Garrido F. Algarra I. García-Lora A. (2010). The escape of cancer from T lymphocytes: immunoselection of MHC class I loss variants harboring structural-irreversible “hard” lesions. Cancer Immunol. Immunother. 59, 1601–1606 10.1007/s00262-009-0716-5 20625726 PMC11029827

[B47] Gomez T. S. Billadeau D. D. (2008). T cell activation and the cytoskeleton: you can’t have one without the other. Adv. Immunol. 97, 1–64 10.1016/S0065-2776(08)00001-1 18501768

[B48] Grybko M. J. Pores-Fernando A. T. Wurth G. A. Zweifach A. (2007). Protein kinase C activity is required for cytotoxic T cell lytic granule exocytosis, but the θ isoform does not play a preferential role. J. Leukoc. Biol. 81, 509–519 10.1189/jlb.0206109 17077164

[B49] Haddad E. K. Wu X. Hammer J. A. Henkart P. A. (2001). Defective granule exocytosis in Rab27a-deficient lymphocytes from Ashen mice. J. Cell Biol. 152, 835–842 10.1083/jcb.152.4.835 11266473 PMC2195776

[B50] Haverstick D. M. Engelhard V. H. Gray L. S. (1991). Three intracellular signals for cytotoxic T lymphocyte mediated killing. Independent roles for protein kinase C, Ca2^+^ influx, and Ca2^+^ release from internal stores. J. Immunol. 146, 3306–3313 2026868

[B51] Herz J. Pardo J. Kashkar H. Schramm M. Kuzmenkina E. Bos E. Wiegmann K. Wallich R. Peters P. J. Herzig S. Schmelzer E. Krönke M. Simon M. M. Utermöhlen O. (2009). Acid sphingomyelinase is a key regulator of cytotoxic granule secretion by primary T lymphocytes. Nat. Immunol. 10, 761–768 10.1038/ni.1757 19525969

[B52] Huse M. Lillemeier B. F. Kuhns M. S. Chen D. S. Davis M. M. (2006). T cells use two directionally distinct pathways for cytokine secretion. Nat. Immunol. 7, 247–255 10.1038/ni1304 16444260

[B53] Isakov N. Altman A. (2002). Protein kinase C θ in T cell activation. Annu. Rev. Immunol. 20, 761–794 10.1146/annurev.immunol.20.100301.064807 11861617

[B54] Jiang K. Zhong B. Gilvary D. L. Corliss B. C. Hong-Geller E. Wei S. Djeu J. Y. (2000). Pivotal role of PI-3 kinase in regulation of cytotoxicity in natural killer cells. Nat. Immunol. 1, 419–425 10.1038/80859 11062502

[B55] Jiang K. Zhong B. Gilvary D. L. Corliss B. C. Vivier E. Hong-Geller E. Wei S. Djeu J. Y. (2002). Syk regulation of phosphoinositide 3-kinase-dependent NK cell function. J. Immunol. 168, 3155–3164 11907067 10.4049/jimmunol.168.7.3155

[B56] Kärre K. Ljunggren H. G. Piontek G. Kiessling R. (1986). Selective rejection of H-2-deficient lymphoma variants suggests alternative immune defence strategy. Nature 319, 675–678 10.1038/319675a0 3951539

[B57] Kasibhlatla S. Genestier L. Green D. R. (1999). Regulation of Fas ligand expression during activation-induced cell death in T lymphocytes via nuclear factor κB. J. Biol. Chem. 274, 987–992 10.1074/jbc.274.2.987 9873041

[B58] Kato H. Takeuchi O. Sato S. Yoneyama M. Yamamoto M. Matsui K. Uematsu S. Jung A. Kawai T. Ishii K. Yamaguchi O. Otsu K. Tsujimura T. Koh C. S. Reis e Sousa C. Matsuura Y. Fujita T. Akira S. (2006). Differential roles of MDA5 and RIG-I helicases in the recognition of RNA viruses. Nature 441, 101–105 10.1038/nature04734 16625202

[B59] KEGGpathway (2011). Natural Killer Cell Mediated Cytotoxicity – Homo sapiens. Available at: http://www.genome.jp/kegg/pathway/hsa/hsa04650.html

[B60] Kelly J. M. Darcy P. K. Markby J. L. Godfrey D. I. Takeda K. Yagita H. Smyth M. J. (2002). Induction of tumor-specific T cell memory by NK cell–mediated tumor rejection. Nat. Immunol. 3, 83–90 10.1038/nrg738 11743585

[B61] Koebel C. M. Vermi W. Swann J. B. Zerafa N. Rodig S. J. Old L. J. Smyth M. J. Schreiber R. D. (2007). Adaptive immunity maintains occult cancer in an equilibrium state. Nature 450, 903–907 10.1038/nature06309 18026089

[B62] Kong K. F. Yokosuka T. Canonigo-Balancio A. J. Isakov N. Saito T. Altman A. (2011). A motif in the V3 domain of the kinase PKC-θ determines its localization in the immunological synapse and functions in T cells via association with CD28. Nat. Immunol. 12, 1105–1112 10.1038/ni.2120 21964608 PMC3197934

[B63] König S. Nimtz M. Scheiter M. Ljunggren H. G. Bryceson Y. T. Jänsch L. (2012). Kinome analysis of receptor-induced phosphorylation in human natural killer cells. PLoS ONE 7, e29672 10.1371/journal.pone.0032145 22238634 PMC3251586

[B64] Kroger N. (2011). Approaches to relapse after allogeneic stem cell transplantation. Curr. Opin. Oncol. 23, 203–208 10.1097/CCO.0b013e328342c6c8 21157340

[B65] Krzewski K. Chen X. Orange J. S. Stromminger J. L. (2006). Formation of a WIP-, WASp-, actin-, and myosin IIA–containing multiprotein complex in activated NK cells and its alteration by KIR inhibitory signaling. J. Cell Biol. 173, 121–132 10.1083/jcb.200509076 16606694 PMC2063796

[B66] Krzewski K. Chen X. Strominger J. L. (2008). WIP is essential for lytic granule polarization and NK cell cytotoxicity. Proc. Natl. Acad. Sci. U.S.A. 105, 2568–2573 10.1073/pnas.0711593105 18258743 PMC2268177

[B67] Krzewski K. Strominger J. L. (2008). The killer’s kiss: the many functions of NK cell immunological synapses. Curr. Opin. Cell Biol. 2008, 597–605 10.1016/j.ceb.2008.05.006 18639449 PMC2577014

[B68] Kuhn J. R. Poenie M. (2002). Dynamic polarization of the microtubule cytoskeleton during CTL-mediated killing. Immunity 16, 111–121 10.1016/S1074-7613(02)00262-5 11825570

[B69] Lanier L. L. (2008). Up on the tightrope: natural killer cell activation and inhibition. Nat. Immunol. 9, 495–502 10.1038/nrm2444 18425106 PMC2669298

[B70] Latinis K. M. Norian L. A. Eliason S. L. Koretzky G. A. (1997). Two NFAT transcription factor binding sites participate in the regulation of CD95 (Fas) ligand expression in activated human T cells. J. Biol. Chem. 272, 31427–31434 10.1074/jbc.272.50.31427 9395475

[B71] Li C. Ge B. Nicotra M. Stern J. N. Kopcow H. D. Chen X. Strominger J. L. (2008). JNK MAP kinase activation is required for MTOC and granule polarization in NKG2D-mediated NK cell cytotoxicity. Proc. Natl. Acad. Sci. U.S.A. 105, 3017–3022 10.1073/pnas.0710905105 18287025 PMC2268577

[B72] Liu R. B. Engels B. Arina A. Schreiber K. Hyjek E. Schietinger A. Binder D. C. Butz E. Krausz T. Rowley D. A. Jabri B. Schreiber H. (2012). Densely granulated murine NK cells eradicate large solid tumors. Cancer Res. 72, 1964–1974 10.1158/0008-5472.CAN-11-3208 22374983 PMC3680344

[B73] Ljunggren H. G. Karre K. (1985). Host resistance directed selectively gainst H-2-deficient lymphoma variants. Analysis of the mechanism. J. Exp. Med. 162, 1745–1795 10.1084/jem.162.6.1745 3877776 PMC2187973

[B74] López-Botet M. Bellón T. Llano M. Navarro F. García P. de Miguel M. (2000). Paired inhibitory and triggering NK cell receptors for HLA class I molecules. Hum. Immunol. 61, 7–17 10.1016/S0198-8859(99)00161-5 10658973

[B75] Marcenaro S. Gallo F. Martini S. Santoro A. Griffiths G. Aricó M. Moretta L. Pende D. (2006). Analysis of natural killer-cell function in familial hemophagocytic lymphohistiocytosis (FHL): defective CD107a surface expression heralds Munc13-4 defect and discriminates between genetic subtypes of the disease. Blood 108, 2316–2323 10.1182/blood-2006-04-015693 16778144

[B76] Melief C. J. M. (2007). Immune pact with the enemy. Nature 450, 803–804 10.1038/nature06363 18026088

[B77] Miller J. S. Soignier Y. Panoskaltsis-Mortari A. McNearney S. A. Yun G. H. Fautsch S. K. McKenna D. Le C. Defor T. E. Burns L. J. Orchard P. J. Blazar B. R. Wagner J. E. Slungaard A. Weisdorf D. J. Okazaki I. J. McGlave P. B. (2005). Successful adoptive transfer and in vivo expansion of human haploidentical NK cells in patients with cancer. Blood 105, 3051–3057 10.1182/blood-2004-07-2974 15632206

[B78] Monks C. R. F. Kupfer H. Tamir I. Barlow A. Kupfer A. (1997). Selective modulation of protein kinase C-θ during T-cell activation. Nature 385, 83–86 10.1038/385083a0 8985252

[B79] Moretta A. Marcenaro E. Parolini S. Ferlazzo G. Moretta L. (2008). NK cells at the interface between innate and adaptive immunity. Cell Death Differ. 15, 226–233 10.1038/sj.cdd.4402170 17541426

[B80] Mustelin T. Coggeshall K. M. Isakov N. Altman A. (1990). T cell antigen receptor-Mediated activation of phospholipase C requires tyrosine phosphorylation. Science 247, 1584–1587 10.1126/science.2138816 2138816

[B81] Nedellec S. Sabourin C. Bonneville M. Scotet E. (2010). NKG2D costimulates human Vγ9Vδ2 T cell antitumor cytotoxicity through protein kinase Cθ-dependent modulation of early TCR-induced calcium and transduction signals. J. Immunol. 185, 55–63 10.4049/jimmunol.1000373 20511557

[B82] Nesic D. Henderson S. Vukmanovic S. (1998). Prevention of antigen-induced MTOC reorientation in cytotoxic T cells by modulation of PKC activity. Int. Immunol. 10, 1741–1746 10.1093/intimm/10.11.1741 9846703

[B83] Nishimura T. Burakoff S. J. Herrmann S. H. (1987). Protein kinase C required for cytotoxic T lymphocyte triggering. J. Immunol. 139, 2888–2891 3499460

[B84] Orange J. Harris K. Andzelm M. Valter M. Geha R. Strominger J. (2003). The mature activating natural killer cell immunologic synapse is formed in distinct stages. Proc. Natl. Acad. Sci. U.S.A. 100, 14151–14156 10.1073/pnas.1835830100 14612578 PMC283561

[B85] O’Rourke A. M. Mescher M. F. (1992). Cytotoxic T-lymphocyte activation involves a cascade of signalling and adhesion events. Nature 358, 253–255 10.1038/358253a0 1630493

[B86] Page K. M. Chaudhary D. Goldman S. J. Kasaian M. T. (2008). Natural killer cells from protein kinase C þeta–/– mice stimulated with interleukin-12 are deficient in production of interferon-γ. J. Leukoc. Biol. 83, 1267–1276 10.1189/jlb.1107745 18263766

[B87] Pardo J. Balkow S. Anel A. Simon M. M. (2002). Granzymes are critically involved in NK-mediated control of RMA-S tumor growth *in vivo*. Eur. J. Immunol. 32, 2881–2886 10.1002/1521-4141(2002010)32:10<2881::AID-IMMU2881>3.0.CO;2-K 12355441

[B88] Pardo J. Buferne M. Martínez-Lorenzo M. J. Naval J. Schmitt-Verhulst A. M. Boyer C. Anel A. (2003). Differential implication of PKC isoforms in TCR/CD3-induced Fas ligand expression and in CTL degranulation. Int. Immunol. 15, 1441–1450 10.1093/intimm/dxg141 14645153

[B89] Pfeifhofer C. Kofler K. Gruber T. Tabrizi N. G. Lutz C. Maly K. Leitges M. Baier G. (2003). Protien kinase C θ affects Ca2^+^ mobilization and NFAT cell activation in primary mouse T cells. J. Exp. Med. 197, 1525–1535 10.1084/jem.20020234 12782715 PMC2193906

[B90] Puente L. G. He J. S. Ostergaard H. L. (2006). A novel PKC regulates ERK activation and degranulation of cytotoxic T lymphocytes: plasticity in PKC regulation of ERK. Eur. J. Immunol. 36, 1009–1018 10.1002/eji.200535277 16552708

[B91] Quann E. J. Liu X. Altan-Bonnet G. Huse M. (2011). A cascade of protein kinase C isozymes promotes cytoskeletal polarization in T cells. Nat. Immunol. 12, 647–654 10.1038/ni.2033 21602810 PMC3119370

[B92] Reefman E. Kay J. G. Wood S. M. Offenhäuser C. Brown D. L. Roy S. Stanley A. C. Low P. C. Manderson A. P. Stow J. L. (2010). Cytokine secretion is distinct from secretion of cytotoxic granules in NK cells. J. Immunol. 184, 4852–4862 10.4049/jimmunol.0803954 20368273

[B93] Robertson L. K. Mireau L. R. Ostergaard H. L. (2005). A role for phosphatidylinositol 3-kinase in TCR-stimulated ERK activation leading to paxillin phosphorylation and CTL degranulation. J. Immunol. 175, 8138–8145 16339552 10.4049/jimmunol.175.12.8138

[B94] Robertson L. K. Ostergaard H. L. (2011). Paxillin associates with the microtubule cytoskeleton and the immunological synapse of CTL through its leucine-aspartic acid domains and contributes to microtubule organizing center reorientation. J. Immunol. 187, 5824–5833 10.4049/jimmunol.1003690 22043013

[B95] Rubnitz J. E. Inaba H. Ribeiro R. C. Pounds S. Rooney B. Bell T. Pui C. H. Leung W. (2010). NKAML: a pilot study to determine the safety and feasibility of haploidentical natural killer cell transplantation in childhood acute myeloid leukemia. J. Clin. Oncol. 28, 955–959 10.1200/JCO.2009.24.4590 20085940 PMC2834435

[B96] Ruggeri L. Mancusi A. Burchielli E. Aversa F. Martelli M. F. Velardi A. (2007a). Natural killer cell alloreactivity in allogeneic hematopoietic transplantation. Curr. Opin. Oncol. 19, 142–147 10.1097/CCO.0b013e3280148a1a 17272987

[B97] Ruggeri L. Mancusi A. Capanni M. Urbani E. Carotti A. Aloisi T. Stern M. Pende D. Perruccio K. Burchielli E. Topini F. Bianchi E. Aversa F. Martelli M. F. Velardi A. (2007b). Donor natural killer cell allorecognition of missing self in haploidentical hematopoietic transplantation for acute myeloid leukemia: challenging its predictive value. Blood 110, 433–440 10.1182/blood-2006-07-038687 17371948 PMC1896125

[B98] Screpanti V. Wallin R. P. A. Ljunggren H. G. Grandien A. (2001). A central role for death receptor-mediated apoptosis in the rejection of tumors by NK cells. J. Immunol. 167, 2068–2073 11489989 10.4049/jimmunol.167.4.2068

[B99] Secrist J. P. Karnitz L. Abraham R. T. (1991). T-cell antigen receptor ligation induces tyrosine phosphorylation of phospholipase C-γ1. J. Biol. Chem. 266, 12135–12139 2061301

[B100] Segovis C. M. Schoon R. A. Dick C. J. Nacusi L. P. Leibson P. J. Billadeau D. D. (2009). PI3K links NKG2D signaling to a CrkL pathway involved in natural killer cell adhesion, polarity, and granule secretion. J. Immunol. 182, 6933–6942 10.4049/jimmunol.0803840 19454690 PMC2706535

[B101] Shibuya A. Lanier L. L. Phillips J. H. (1998). Protein kinase C is involved in the regulation of both signaling and adhesion mediated by DNAX accessory molecule-1 receptor. J. Immunol. 161, 1671–1676 9712030

[B102] Smyth M. J. Kelly J. M. Baxter A. G. Körner H. Sedgwick J. D. (1998). An essential role for tumor necrosis factor in natural killer cell–mediated tumor rejection in the peritoneum. J. Exp. Med. 188, 1611–1619 10.1084/jem.188.9.1611 9802973 PMC2212521

[B103] Smyth M. J. Thia K. Y. T. Street S. E. A. Cretney E. Trapani J. A. Taniguchi M. Kawano T. Pelikan S. B. Crowe N. Y. Godfrey D. I. (2000). Differential tumor surveillance by natural killer (NK) and NKT cells. J. Exp. Med. 191, 661–668 10.1084/jem.191.4.661 10684858 PMC2195840

[B104] Sorkin L. S. Otto M. Baldwin W. M. Vail E. Gillies S. D. Handgretinger R. Barfield R. C. Ming Yu H. Yu A. L. (2010). Anti-GD(2) with an Fc point mutation reduces complement fixation and decreases antibody-induced allodynia. Pain 149, 135–142 10.1016/j.pain.2010.01.024 20171010 PMC3755890

[B105] Stern M. Ruggeri L. Mancusi A. Bernardo M. E. de Angelis C. Bucher C. Locatelli F. Aversa F. Velardi A. (2008). Survival after T cell-depleted haploidentical stem cell transplantation is improved using the mother as donor. Blood 112, 2990–2995 10.1182/blood-2008-01-135285 18492955 PMC2962448

[B106] Stinchcombe J. C. Barral D. C. Mules E. H. Booth S. Hume A. N. Machesky L. M. Seabra M. C. Griffiths G. M. (2001a). Rab27a is required for regulated secretion in cytotoxic T lymphocytes. J. Cell Biol. 152, 825–834 10.1083/jcb.152.4.825 11266472 PMC2195783

[B107] Stinchcombe J. C. Bossi G. Booth S. Griffiths G. M. (2001b). The immunological synapse of CTL contains a secretory domain and membrane bridges. Immunity 15, 751–761 10.1016/S1074-7613(01)00234-5 11728337

[B108] Stinchcombe J. C. Griffiths G. M. (2007). Secretory mechanisms in cell-mediated cytotoxicity. Annu. Rev. Cell Dev. Biol. 23, 495–517 10.1146/annurev.cellbio.23.090506.123521 17506701

[B109] Sun Z. Arendt C. W. Ellmeier W. Schaeffer E. M. Sunshine M. J. Gandhi L. Annes J. Petrzilka D. Kupfer A. Schwartzberg P. L. Littman D. R. (2000). PKC-θ is required for TCR-induced NF-κB activation in mature but not in immature T lymphocytes. Nature 404, 402–407 10.1038/35006090 10746729

[B110] Takayama H. Sitkovsky M. V. (1987). Antigen receptor-regulated exocytosis in cytotoxic T lymphocytes. J. Exp. Med. 166, 725–743 10.1084/jem.166.3.725 2442289 PMC2188687

[B111] Tassi I. Cella M. Presti R. Colucci A. Gilfillan S. Littman D. R. Colonna M. (2008). NK cell activating receptors require PKCθ for sustained signaling, transcriptional activation and IFN-γ secretion. Blood 112, 4109–4116 10.1182/blood-2008-02-139527 18784374 PMC2581989

[B112] Terme M. Ullrich E. Delahaye N. F. Chaput N. Zitvogel L. (2008). Natural killer cell–directed therapies: moving from unexpected results to successful strategies. Nat. Immunol. 9, 486–494 10.1038/ni1580 18425105

[B113] Upshaw J. L. Arneson L. N. Schoon R. A. Dick C. J. Billadeau D. D. Leibson P. J. (2006). NKG2D-mediated signaling requires a DAP10-bound Grb2-Vav1 intermediate and phosphatidylinositol-3-kinase in human natural killer cells. Nat. Immunol. 7, 524–532 10.1038/nrg1893 16582911

[B114] Van den Broek M. F. Kägi D. Zinkernagel R. M. Hengartner H. (1995). Perforin dependence of natural killer cell-mediated tumor control *in vivo*. Eur. J. Immunol. 25, 3514–3516 10.1002/eji.1830251246 8566046

[B115] Velardi A. (2008). Role of KIRs and KIR ligands in hematopoietic transplantation. Curr. Opin. Immunol. 20, 581–587 10.1016/j.coi.2008.07.004 18675345

[B116] Villalba M. Bi K. Hu J. Altman Y. Bushway P. Reits E. Neefjes J. Baier G. Abraham R. T. Altman A. (2002). Translocation of PKCθ in T cells is mediated by a nonconventional, PI3-K- and Vav-dependent pathway, but does not absolutely require phospholipase C. J. Cell Biol. 157, 253–263 10.1083/jcb.200201097 11956228 PMC2199257

[B117] Villalba M. Bi K. Rodriguez F. Tanaka Y. Schoenberger S. Altman A. (2001a). Vav1/Rac-dependent actin cytoskeleton reorganization is required for lipid raft clustering in T cells. J. Cell Biol. 155, 331–338 10.1083/jcb.200107080 11684704 PMC2150846

[B118] Villalba M. Bushway P. Altman A. (2001b). PKCθ mediates a selective T cell survival signal via phosphorylation of BAD. J. Immunol. 166, 5955–5963 11342610 10.4049/jimmunol.166.10.5955

[B119] Villalba M. Kasibhatla S. Genestier L. Mahboubi A. Green D. R. Altman A. (1999). Protein kinase C-θ cooperates with calcineurin to induce Fas ligand expression during activation-induced T cell death. J. Immunol. 163, 5813–5819 10570264

[B120] Villunger A. Ghaffari-Tabrizi N. Tinhofer I. Krumböck N. Bauer B. Schneider T. Kasibhatla S. Greil R. Baier-Bitterlich G. Überall F. Green D. R. Baier G. (1999). Synergistic action of protein kinase C θ and calcineurin is sufficient for Fas ligand expression and induction of a CrmA-sensitive apoptosis pathway in Jurkat T cells. Eur. J. Immunol. 29, 3549–3561 10.1002/(SICI)1521-4141(199911)29:11<3549::AID-IMMU3549>3.0.CO;2-Q 10556809

[B121] Vivier E. Tomasello E. Baratin M. Walzer T. Ugolini S. (2008). Functions of natural killer cells. Nat. Immunol. 9, 503–510 10.1038/ni1582 18425107

[B122] Vosshenrich C. A. J. Ranson T. Samson S. I. Corcuff E. Colucci F. Rosmaraki E. E. DiSanto J. P. (2005). Roles for common cytokine receptor γ-chain-dependent cytokines in the generation, differentiation, and maturation of NK cell precursors and peripheral NK cells *in vivo*. J. Immunol. 174, 1213–1221 15661875 10.4049/jimmunol.174.3.1213

[B123] Vyas Y. Maniar H. Dupont B. (2002a). Visualization of signaling pathways and cortical cytoskeleton in cytolytic and noncytolytic natural killer cell immune synapses. Immunol. Rev. 189, 161–178 10.1034/j.1600-065X.2002.18914.x 12445273

[B124] Vyas Y. M. Maniar H. Dupont B. (2002b). Differential segregation of the SRC homology 2-containing protein tyrosine phosphatase-1 within the early NK cell immune synapse distinguishes noncytolytic from cytolytic interactions. J. Immunol. 168, 3150–3154 11907066 10.4049/jimmunol.168.7.3150

[B125] Willemze R. Rodrigues C. A. Labopin M. Sanz G. Michel G. Socie G. Rio B. Sirvent A. Renaud M. Madero L. Mohty M. Ferra C. Garnier F. Loiseau P. Garcia J. Lecchi L. Kögler G. Beguin Y. Navarrete C. Devos T. Ionescu I. Boudjedir K. Herr A. L. Gluckman E. Rocha V. Eurocord-Netcord and Acute Leukaemia Working Party of the EBMT (2009). KIR-ligand incompatibility in the graft-versus-host direction improves outcomes after umbilical cord blood transplantation for acute leukemia. Leukemia 23, 492–500 10.1038/leu.2009.11 19151783 PMC7101531

[B126] Zeng J. Cannon J. L. Abraham R. T. Way M. Billadeau D. D. Bubeck-Wardenberg J. Burkhardt J. K. (2003). SLP-76 coordinates Nck-dependent WASP recruitment with Vav-1/Cdc42-dependent WASP activation at the T cell–APC contact site. J. Immunol. 171, 1360–1368 12874226 10.4049/jimmunol.171.3.1360

